# Stimulation of Osteoclast Formation by RANKL Requires Interferon Regulatory Factor-4 and Is Inhibited by Simvastatin in a Mouse Model of Bone Loss

**DOI:** 10.1371/journal.pone.0072033

**Published:** 2013-09-11

**Authors:** Yoshiki Nakashima, Tatsuji Haneji

**Affiliations:** Department of Histology and Oral Histology, Institute of Health Biosciences, The University of Tokushima Graduate School, Tokushima, Japan; The University of Tennessee Health Science Center, United States of America

## Abstract

Diseases of bone loss are a major public health problem. Here, we report the novel therapeutic action of simvastatin in osteoclastogenesis and osteoprotection, demonstrated by the ability of simvastatin to suppress osteoclast formation *in vitro* and *in vivo*. We found that *in vitro*, IRF4 expression is upregulated during osteoclast differentiation induced by RANKL (receptor activator of nuclear factor-κB ligand), while simvastatin blocks RANKL-induced osteoclastogenesis and decreases expression of NFATc1 (nuclear factor of activated T-cells, cytoplasmic, calcineurin-dependent 1), IRF4 and osteoclast markers. We also show that IRF4 acts in cooperation with NFATc2 and NF-κB on the promoter region of NFATc1 to accelerate its initial transcription during the early stage of osteoclastogenesis. Moreover, our study using IRF4 siRNA knockdown directly demonstrates the requirement for IRF4 in NFATc1 mRNA transcription and its necessity in RANKL-induced osteoclast differentiation. Our results suggest that the reduction in osteoclastogenesis is partly due to the inhibition of IRF4 production in RANKL-induced osteoclast differentiation. To investigate the *in vivo* effects of simvastatin in RANKL-treated mice, we examined the bone mineral density (BMD) of a mouse model of bone loss, and found that simvastatin significantly reduced bone loss by suppressing osteoclast numbers *in vivo*, even in the presence of high concentrations of RANKL. These results suggest that the depletion of osteoclasts is not due to the reduction in RANKL produced by osteoblasts *in vivo*. The results are consistent with the hypothesis that simvastatin blocks RANKL-induced IRF4 expression in osteoclastogenesis. We propose that the expression of IRF4 by osteoclasts could be a promising new therapeutic target in bone-loss diseases.

## Introduction

RANKL/RANK signaling induces osteoclast formation and activation via several transcription factors, such as interferon-regulatory factors (IRFs) [1], [2], c-Fos, NF-κB and NFATc1 [3], [4]. It has also been shown that NFATc1 cooperates with PU.1 on the *Cathepsin K* and *OSCAR* promoters [5], [6], and forms an osteoclast-specific transcriptional complex containing AP-1 (Fos/Jun) and PU.1 for the efficient induction of osteoclast-specific genes, such as *Atp6v0d2*, *Cathepsin K*, *DC-STAMP* and *TRAP* [4], [7], [8]. PU.1 confers specificity to the NFATc1 response in RAW264.7 cells [9]. IRF4 and interferon consensus sequence-binding protein (ICSBP)/IRF8 are members of the IRF family, which are expressed in bone marrow-derived cells [10]. Both factors can be recruited to the IRF DNA-binding site in target genes through interaction with PU.1 [11]–[14]. Recently, an *in vivo* and *in vitro* study indicated that IRF8 suppresses osteoclastogenesis. In osteoclast precursors, abundant IRF8 interacts with basally-expressed NFATc1 to suppress its transcriptional activity and thus prevent its activation of target genes, including autoamplification of its own promoter [15]. However, our understanding of the function of IRF4 in osteoclastogenesis remains elusive. Therefore, in this study, to dissect further these IRF4 functions in osteoclast differentiation, we focused on the transcriptional control of NFATc1 gene expression in RAW264.7 cells. Furthermore, we performed a pharmacological experiment to identify inhibitors of IRF4. Simvastatin is an orally administered competitive inhibitor of 3-hydroxy-3-methyl-glutaryl-coenzyme A (HMG-CoA) reductase, an enzyme that catalyzes the conversion of HMG-CoA to mevalonic acid [16]. As effective cholesterol-lowering agents, statins have been extensively used for prevention of cardiovascular disease. Simvastatin inhibits the isoprenoids farnesyl pyrophosphate and geranylgeranyl pyrophosphate (GGPP). These isoprenoid pyrophosphates serve as essential adjuncts in the post-translational modification of numerous key proteins that function as molecular switches, including the small GTPases RAS, RAC and RAS homologue (RHO) [17], [18]. Osteoclast survival, differentiation and function require the GTPases including RAS [19]–[21], RAC [22], [23] and RHO [24], [25]. The membrane attachment and biological activity of these small GTPases require prenylation. The Rho family of GTPases is a large family of proteins, which includes RhoA, Rac1 and Rac2. Rho kinase (ROCK) has been shown to activate the DNA binding of IRF4 [26], while another report showed that simvastatin inhibits IRF4 gene expression via selective inhibition of ROCK in Th17 cells [27]. Therefore, in this study, we used simvastatin as an inhibitor of IRF4, and report the role of IRF4 in osteoclast differentiation in the presence of RANKL. Our study shows that IRF4 is a constituent of the signalling pathways that mediate the effect of prenylated GTPases on RANK/RANKL-dependent osteoclastogenesis *in vitro* and *in vivo*.

## Materials and Methods

### Reagents

Reagents were obtained from the following suppliers: Alpha-modified Minimum Essential Medium (α-MEM): Invitrogen (Carlsbad, CA). Fetal bovine serum (FBS): MBL (Nagoya, Japan). Recombinant mouse RANKL: Oriental Yeast Co., Ltd. (Shiga, Japan). Simvastatin: Tokyo Chemical Industry co., (Tokyo, Japan). Y-27632: WAKO (Osaka, Japan). BAY11-7082: Gentaur (Kampenhout, Belgium). Anti-β-actin antibody: Sigma-Aldrich (St. Louis, MO). Anti-B23 (C-19), anti-Eps15 (C-20), anti-IRF4 (M-17), anti-IRF8 (C-19), anti-NFATc1 (7A6), anti-NFATc2 (4G6-G5), anti-NF-κB p65 (C-20) and anti-TRAP (K-17) antibodies: Santa Cruz Biotechnology (Santa Cruz, CA). Anti-EZH2 (AC22) antibodies: Cell Signaling Technology (Boston, MA). Anti-osteopontin (O-17) antibody: Immuno-Biological Laboratories Co., Ltd. (Gunma, Japan). Plastic dishes: IWAKI (Chiba, Japan).

### Animal care

All experimental protocols were in accordance with the guidelines for the care and use of laboratory animals set by the Graduate School of the Institute of Health Biosciences, the University of Tokushima (Tokushima, Japan). The protocol was approved by the Committee on Animal Experiments of the University of Tokushima (permit number: 12052 and 12067). C57BL/6J female mice (4–8 weeks old; Japan SLC, Shizuoka, Japan) were maintained under controlled temperature (23±2°C) and light conditions (lights on from 08:30–20:30) and fed standard rodent chow pellets with water *ad libitum*. All efforts were made to minimize the suffering of the animals.

### Animal treatment

To evaluate the effect of chronic administration of the drug, simvastatin (10 mg/kg) or saline was injected intraperitoneally into 4-week-old female mice (n = 5/group) at 24-h intervals for 4 weeks before sacrifice.

A mouse model of bone loss was established as described [28]. Briefly, RANKL (1 mg/kg) or saline was injected intraperitoneally into 7-week-old female mice (n = 5/group). After 48 h the mice were killed and the femora were harvested for analysis. To evaluate the effect of simvastatin on this model of bone loss, simvastatin (10 mg/kg) was injected intraperitoneally 24 h before the first RANKL injection, followed by simvastatin injections at 24-h intervals for 2 days before sacrifice (n = 5).

### Bone densitometry

Femora were harvested for μCT analysis. Tomographic measurements of bone mineral density (BMD) and bone densitometry were analysed on an animal CT system (LaTheta LCT-100; Aloka, Tokyo, Japan) using voxel size of 24×24×24 µm^3^. BMD (milligrams per cubic centimetre) was calculated using LaTheta software (version 1.00). Radiographic tomography was constructed using high-feature software (OsiriX v.4.1.2 64-bit).

### Cell Culture

RAW264.7 cells (mouse macrophage-derived cells, purchased from RIKEN Cell Bank) were cultured in plastic dishes containing α-MEM supplemented with 10% FBS in a CO_2_ incubator (5% CO_2_ in air) at 37°C and subcultured every 2 days.

### Cell differentiation assays

For osteoclastic differentiation, RAW264.7 cells were seeded into 96-well plates at 2,000 cells/150 µL of α-MEM containing 10% FBS and 50 ng/mL RANKL (‘osteoclastogenic medium’). The medium was changed every 2nd day. TRAP staining was as described previously [29].

### Real time PCR and RT-PCR

Cells were cultured in 35 mm dishes in osteoclastogenic medium to ∼80% confluence. RNA preparation, real time PCR analyses and RT-PCR analyses were as described previously [30], [31], and were performed using primers listed in Table 1. Images were recorded using an ATTO CS analyser (ATTO, Tokyo, Japan).

**Table 1:** Sequences of quantitative PCR primers.

List of primers used for Real time PCR and RT-PCR		
Genes	Forward primer	Reverse primer
Atp6v0d2	TCAGATCTCTTCAAGGCTGTGCTG	GTGCCAAATGAGTTCAGAGTGATG
cathepsin K	CACCCAGTGGGAGCTATGGAA	GCCTCCAGGTTATGGGCAGA
DC-STAMP	AAACGATCAAAGCAGCCATTGAG	ATCATCTTCATTTGCAGGGATTGTC
IRF4	CAAAGCCCTCAGTCGTTGTCC	TCTGTGCTCCAATCCCAGAGTG
jmjd3	CTGCTGTAACCCACTGCTGGA	GAAAGCCAATCATCACCCTTGTC
NFATc1	TGGAGAAGCAGAGCACAGAC	GCGGAAAGGTGGTATCTCAA
TRAP	ACCTTGGCAACGTCTCTGCAC	CTCCAGCATAAAGATGGCCACA
GAPDH	AAATGGTGAAGGTCGGTGTG	TGAAGGGGTCGTTGATGG

List of primers used for ChIP Assay		
Genes	Forward primer	Reverse primer
IRF4 promoter	CCAGAACCCAGGATGGAAGA	GGTCAACTTGGAGCGTTTGTAAA
NFATc1 promoter	CCGGGACGCCCATGCAATCTGTTAGTAATT	GCGGGTGCCCTGAGAAAGCTACTCTCCCTT

### Western blotting analysis

RAW264.7 cells were cultured in 60 mm dishes in osteoclastogenic medium to ∼80% confluence. Western blotting analysis was as described previously [32]. Blots were probed using specific antibodies for B23, EPS, EZH2, IRF4, Jmjd3, NFATc1, NFATc2, NF-κB p65 or β-actin. Images were quantified using National Institutes of Health (NIH) Image J software (Version 1.44; http://imagej.nih.gov/ij).

### Immunohistochemistry

Tissues were fixed in 4% paraformaldehyde, decalcified in 2.5% EDTA (pH 7.2) containing 0.4 M glucose at 4°C for 2 weeks, dehydrated and embedded in paraffin. Antigens were retrieved with 0.4 mg/mL proteinase K at room temperature for 5 min. After quenching of endogenous peroxidase activity with 1% H_2_O_2_ in methanol, sections were incubated with an anti-TRAP polyclonal antibody (Santa Cruz Biotechnology) or anti-osteopontin antibody: (Immuno-Biological Laboratories Co., Ltd.) at 4°C overnight, washed with PBS, then incubated with peroxidase-conjugated secondary antibody according to the manufacturer's instructions (Histofine Simple Stain MAX-PO, Nichirei Bioscience). Colour was developed with 3,3-diaminobenzidine tetrahydrochloride (DAB), and haematoxylin was used as a nuclear counterstain.

### Immunoprecipitation

RAW264.7 cells were cultured in 100 mm dishes in osteoclastogenic medium to ∼80% confluence. Immunoprecipitation was performed as described previously [33], using specific antibodies for IRF4 and IRF8.

### Chromatin Immunoprecipitation (ChIP) Assay

RAW264.7 cells were cultured in 100 mm dishes in osteoclastogenic medium to ∼80% confluence. The Chip Assay was described previously [34]. DNA was extracted with a Wizard Genomic DNA Purification Kit (Promega KK, Tokyo, Japan). Ethanol-precipitated DNA was solubilized in water (1.0×10^6^ cell equivalent/30 µL). Semiquantitative PCR was performed following the method for RT-PCR, and was performed using primers listed in Table 1.

### siRNA knockdown

siRNAs, chemically synthesized and purified by HPLC, were purchased from Japan Bio Services Co., Ltd. (Saitama, Japan). siRNA were performed using sequences listed in Table 2.

**Table 2:** Sequences of siRNA duplexes.

List of siRNA sequences		
Genes	Forward primer	Reverse primer
IRF4	GCAUGUUUUAGUUUUCAAUTT	AUUGAAAACUAAAACAUGCTT
nontargeting control	UCCUAUAUAUGUUUGUAGUTT	ACUACAAACAUAUAUAGGATT

Transient transfection with siRNA was performed at 2-day intervals in fresh osteoclastogenic medium with HilyMAX reagent (Dojindo, Kumamoto, Japan) in accordance with the manufacturer's instructions.

### Statistical analysis

Statistical analysis was performed using Student's t-test to compare two samples. Statistical analysis of comparisons between multiple groups (more than two groups) was performed using one-way and two-way ANOVA with StatPlus software (AnalystSoft). Statistical significance was set at *P*<0.05 for all tests. Results shown are representative examples of three independent experiments.

## Results

### IRF4 increases during osteoclastogenesis

**Figure 1:**
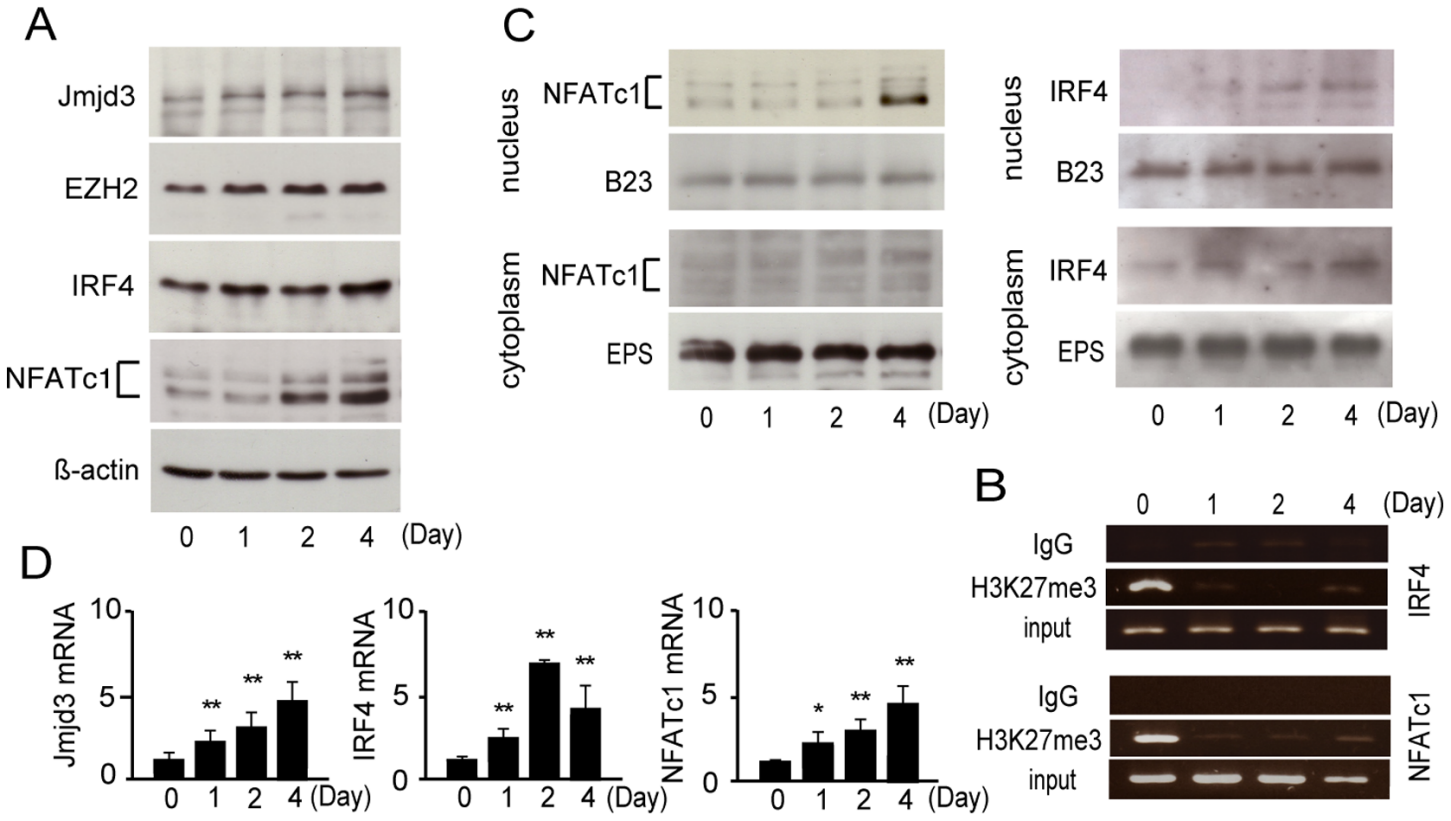
Expression of IRF4 in osteoclastogenesis. (A) Western blot analysis of Jmjd3, EZH2, IRF4, NFATc1 and β-actin protein expression in RAW264.7 cells cultured in the presence of 50 ng/mL RANKL at 0, 1, 2 and 4 d. β-actin served as the loading control. (B) ChIP assay of the IRF4 and NFATc1 promoter region in RAW264.7 cells cultured in the presence of 50 ng/mL RANKL at 0, 1, 2 and 4 d. (C) Western blot analysis of NFATc1 and IRF4 protein expression in nuclear and cytoplasmic fractions of RAW264.7 cells cultured in the presence of 50 ng/mL RANKL at 0, 1, 2 and 4 d. Expression levels of B23 and EPS protein were measured as the loading controls for nuclear and cytoplasmic fractions, respectively. (D) Quantitative real-time PCR analysis of Jmjd3, IRF4 and NFATc1 mRNA in RAW264.7 cells cultured in the presence of 50 ng/mL RANKL at 0, 1, 2 and 4 d. Data represent mean ± S.D. * *P*<0.05, **P<0.01.

To assess the expression of IRF4 during osteoclastogenesis, we used RT-PCR and immunoblot analyses to detect IRF4 expression in RAW264.7 cells after RANKL stimulation (Fig. 1A, D; full-length blots in Fig. S1A), and showed that robust induction of NFATc1 by RANKL is a necessary and pivotal step for osteoclast differentiation characterized by enhanced expression of Jmjd3, which is an H3K27me3 demethylase. In concert, both IRF4 and NFATc1 expression were higher after RANKL stimulation. In addition, activation of EZH2-mediated H3K27 methylation increased during the later stage of osteoclastogenesis (Fig. 1A).

### Epigenetic regulation of IRF4 and NFATc1 genes in osteoclastogenesis

We examined the mechanism underlying the increase in IRF4 and NFATc1 expression with RANKL. We employed a chromatin immunoprecipitation assay using anti-H3K27me3 antibody to evaluate the interaction between H3K27me3-modified DNA with the IRF4 and NFATc1 promoters in RAW264.7 cells. We confirmed by ChIP analysis that H3K27 in the promoter region of IRF4 is methylated in osteoclast precursors (Fig. 1B; full-length gels in Fig. S1B). Another study has indicated that NFATc1 is apparently epigenetically regulated by Jmjd3 in osteoclastogenesis [35], [36]. Furthermore, the expression of both NFATc1 and IRF4 increase with demethylase activity (Fig. 1A, D). NFATc1 binds to its own promoter, which leads to the robust induction of NFATc1 and this autoamplification is critical for osteoclastogenesis. Fig. 1B shows that EZH2-mediated H3K27 methylation of the promoter regions of IRF4 and NFATc1 increases during the later stage of osteoclastogenesis. We consider that the methylation acts to reduce IRF4 gene activation by the second day after RANKL stimulation.

### Nuclear translocation of IRF4 and NFATc1 in osteoclastogenesis

RANKL stimulation resulted in substantially higher concentrations of nuclear IRF4 and NFATc1 protein after 4 days (Fig. 1C; full-length blots in Fig. S1C).

### IRF4 accelerates transcriptional activity of NFATc1

**Figure 2:**
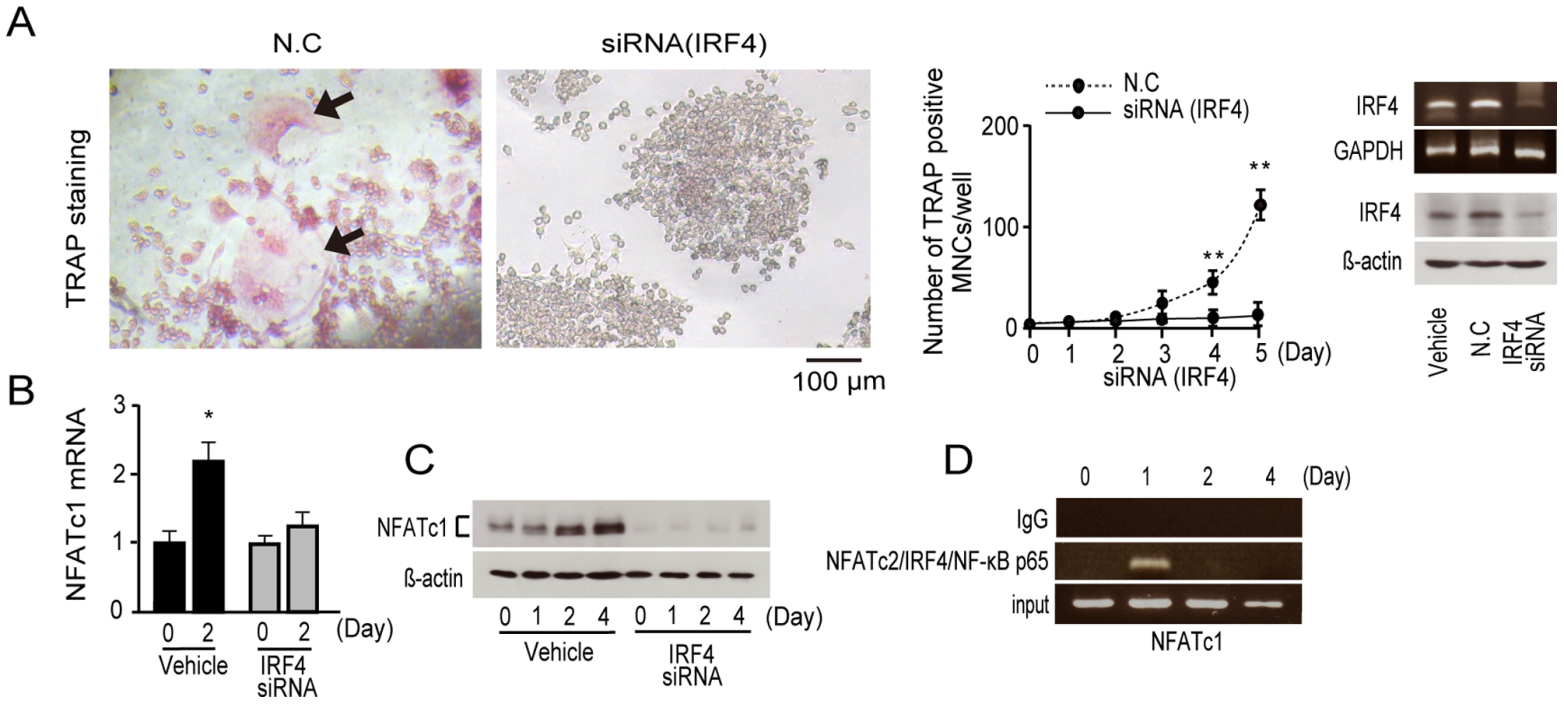
NFATc1 expression in osteoclastogenesis after treatment with IRF4 siRNA. (A) RAW264.7 cells were transfected with IRF4 siRNA or nontargeting control siRNAs (N.C) in the presence of 50 ng/mL RANKL for 5 d. Expression of IRF4 mRNA (top right) and protein (lower right). Expression levels of GAPDH mRNA and β-actin protein were measured as the loading controls. Numbers of TRAP-positive multinucleated cells (MNCs) were counted (middle). TRAP-positive cells appear red in the photomicrograph (left); n = 8. Data represent mean ± S.D. **P<0.01. Scale bar = 100 µm. (B) Quantitative real-time PCR analysis of NFATc1 mRNA in RAW264.7 cells cultured in the presence of 50 ng/mL RANKL and IRF4 siRNA at 2 d; n = 4. * *P*<0.05. (C) Western blot analysis of NFATc1 protein in RAW264.7 cells cultured in the presence of 50 ng/mL RANKL and IRF4 siRNA at 0, 1, 2 and 4 d. β-actin served as the loading control. (D) ChIP analysis of the NFATc1 promoter region in RAW264.7 cells cultured in the presence of 50 ng/mL RANKL at 0, 1, 2 and 4 d.

IRF4-specific siRNA was prepared, and IRF4 knockdown cells were treated with RANKL. We found that IRF4 siRNA markedly suppressed RANKL-induced osteoclast formation (Fig. 2A). The siRNA knockdown was confirmed by attenuated levels of both IRF4 mRNA and protein (Fig. 2A; full-length blots and gels in Fig. S2A). Real-time PCR and western blot analyses confirmed that both NFATc1 mRNA (Fig. 2B) and protein (Fig. 2C; full-length blots in Fig. S2C) were suppressed in osteoclastogenesis. Previous studies showed that cooperation of NFATc2 and NF-κB activates the initial induction of NFATc1 [37]. In addition, our study shows that IRF4 participates in the cooperation of NFATc2 and NF-κB to activate the initial induction of NFATc1 (Fig. 2D; full-length gels in Fig. S2D), which may play a role in early osteoclastogenesis.

### Simvastatin represses osteoclastogenesis by reducing expression of several osteoclast-specific genes

**Figure 3:**
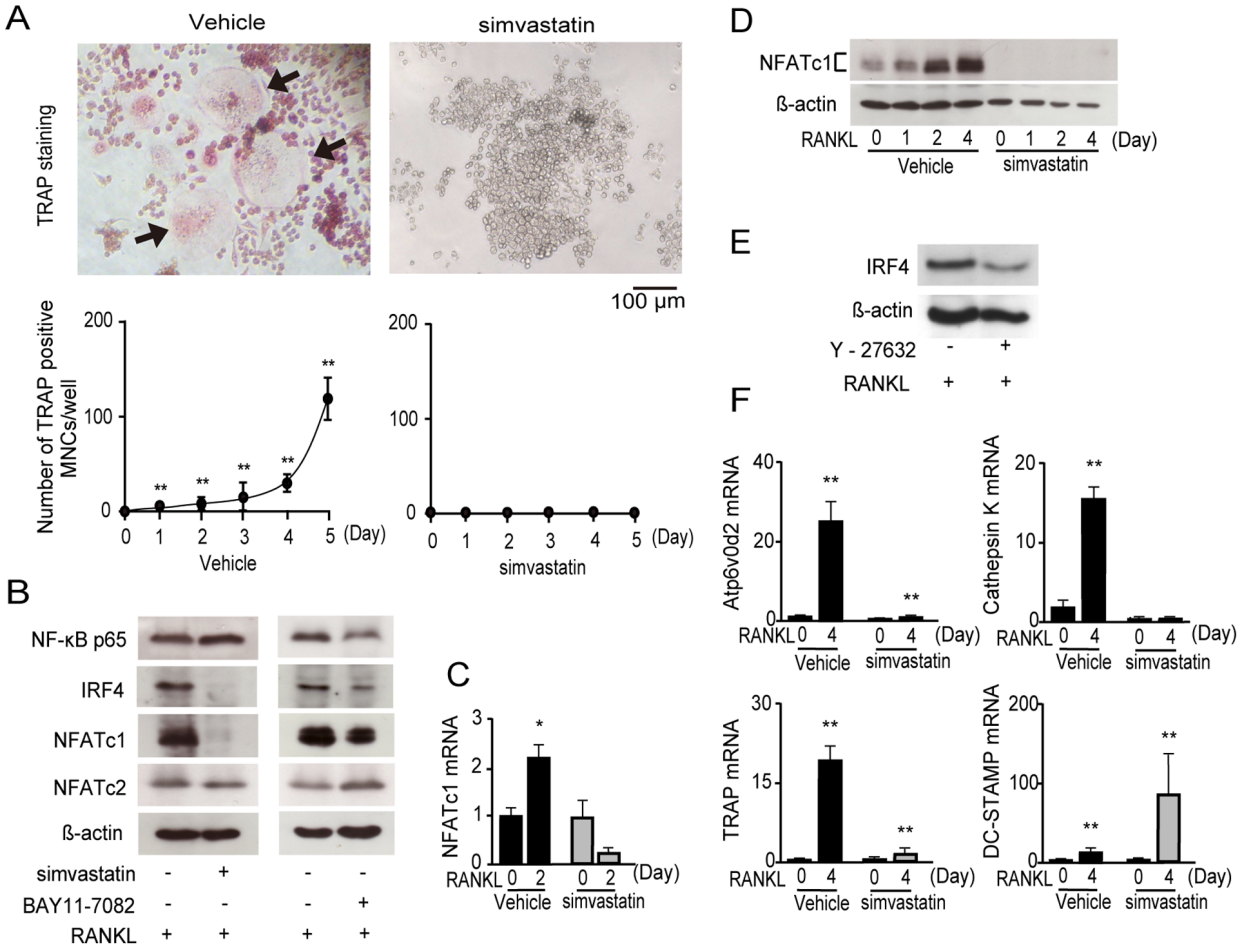
Simvastatin inhibits osteoclastogenesis. (A) RAW264.7 cells cultured in the presence of 50 ng/mL RANKL and 2.5 µM simvastatin for 5 days, stained for TRAP. Top, TRAP-positive cells appear red in the photomicrograph. Black arrows indicate multinucleated osteoclasts. Bottom, TRAP-positive multinucleated cells were counted as osteoclasts; n = 8. Data represent mean ± S.D. * *P*<0.05, **P<0.01. Scale bar = 100 µm. (B) Western blot analysis of NF-κB p65, IRF4, NFATc1, NFATc2 and β-actin proteins in RAW264.7 cells cultured in the presence of 50 ng/mL RANKL, 2.5 µM simvastatin and 5 µM BAY11-7082 at 4 d. β-actin served as the loading control. (C) Quantitative real-time PCR analysis of NFATc1 mRNA in RAW264.7 cells cultured in the presence of 50 ng/mL RANKL and 2.5 µM simvastatin at 2 d; n = 4. Data represent mean ± S.D. * *P*<0.05. (D) Western blot analysis of NFATc1 protein in RAW264.7 cells cultured in the presence of 50 ng/mL RANKL and 2.5 µM simvastatin at 0, 1, 2 and 4 d. β-actin served as the loading control. (E) Western blot analysis of IRF4 protein in RAW264.7 cells cultured in the presence of 50 ng/mL RANKL and 100 µM Y-27632 at 4 d. β-actin served as the loading control. (F) Quantitative real-time PCR analysis of *Atp6v0d2*, *Cathepsin K*, *TRAP* and *DC-STAMP* expression in RAW264.7 cells cultured in the presence of 50 ng/mL RANKL and 2.5 µM simvastatin at 0 and 4 d. n = 5. Data represent mean ± S.D. **P<0.01.

Next, we examined the previously unexplored effect of simvastatin on osteoclast differentiation *in vitro* and *in vivo*. In this study, simvastatin inhibited RANKL-induced osteoclast formation (Fig. 3A). Real-time PCR and western blot analyses confirmed that NFATc1 mRNA (Fig. 3C), IRF4 and NFATc1 protein were suppressed during simvastatin stimulation. The NF-κB inhibitor BAY11-7082 reduced the protein level of both IRF4 and NFATc1 (Fig. 3B, D; full-length blots in Fig. S3B, D). This result shows that the role of IRF4 is partly dependent on NF-κB activation in RANKL-induced osteoclast formation. In addition, we treated RAW264.7 cells with the Rho kinase/ROCK signaling inhibitor Y-27632 and found that IRF4 expression decreased after 4 days of RANKL treatment (Fig. 3E; full-length blots in Fig. S3E). RANKL-stimulated induction of the osteoclastic genes *Atp6v0d2*, *Cathepsin K* and *TRAP* was also severely impaired by simvastatin without affecting the expression of *DC-STAMP* (Fig. 3F).

### *In vivo* effects of simvastatin on bone anomalous absorption

**Figure 4:**
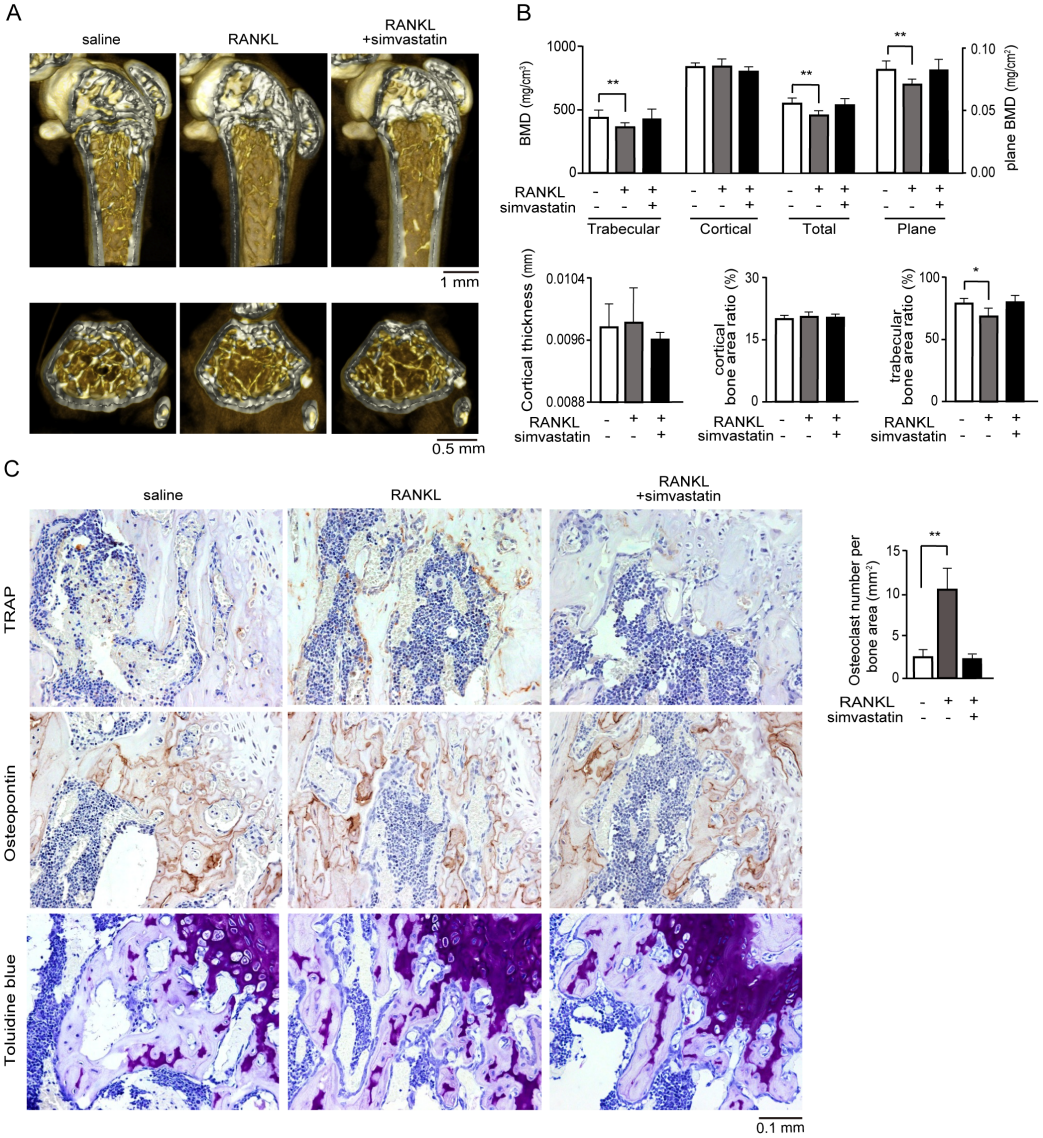
*In vivo* effects of simvastatin in a mouse model of bone loss. (A) 3D images of the distal femur showing the protection of bone mass by simvastatin in mice injected with 1 mg/kg RANKL. Upper panels: sagittal plane; lower panels: transverse plane. (B) Trabecular, cortical, total and plane BMD were measured; n = 5. Data represent mean ± S.D. **P<0.01. Bottom, cortical thickness, cortical bone area ratio and trabecular bone area ratio were measured; n = 5. Data represent mean ± S.D. **P<0.01. (C) Left, TRAP and osteopontin immunostaining, and toluidine blue staining of the distal femur showing inhibition of osteoclast differentiation by 10 mg/kg simvastatin in 1 mg/kg RANKL-injected mice. Right, osteoclast numbers were counted; n = 5. Data represent mean ± S.D. **P<0.01. Scale bar = 0.1 mm.

To prepare a mouse model of bone loss, RANKL was injected intraperitoneally into 7-wk-old female mice. Simvastatin was injected from 1 day before the first RANKL injection. To determine the impact of simvastatin on bone resorption, we performed high-resolution microcomputed tomography (μCT) studies, which showed that simvastatin significantly reduced RANKL-induced bone loss (Fig. 4A, B). This reduction in bone loss was not as evident in the cortical region. The rapid decrease in BMD in this model seems not only to be caused by stimulation of the final differentiation of osteoclast progenitors but also by the activation of a preexisting pool of osteoclasts. We believe that osteoclast precursors are more abundant in the bone marrow than in blood. Bone sections immunostained for tartrate-resistant acid phosphatase (TRAP) revealed that simvastatin significantly reduced the numbers of osteoclasts in bone loss model mice following intraperitoneal administration of RANKL. Osteopontin develops early in bone formation that expression is high during remodeling site and is concerned with the bone morphogenetic process. We observed increases in both bone formation and osteoblastic activity. Immunostaining for osteopontin revealed that simvastatin does not affect bone remodeling activity, while toluidine blue staining revealed a normal rate of new bone formation rate in bone loss model mice following intraperitoneal administration of RANKL.

## Discussion

A clinical trial of simvastatin in postmenopausal female patients with osteoporosis [38], [39] demonstrated the ability of simvastatin to increase new bone formation [40], while an *in vitro* study characterized the mechanisms through which simvastatin (2.5 µM) increases expression of the BMP-2 gene in bone cells [40]. Mundy and colleagues reported [40] increased trabecular bone volume in ovariectomised rats given simvastatin at a daily dose of 5–10 mg/kg for 35 days. Although the dose per body weight in the rats was higher than the lipid-lowering dose used in humans, Mundy and colleagues predicted that there would be similar effects on bone formation in humans at lipid-lowering doses. However the U.S. Food and Drug Administration (FDA) is recommending limiting the use of the highest approved dose of simvastatin (80 mg) because of the increased risk of muscle damage reported in 2011 [41].

Several animal models have been created for the study of bone loss, such as ovariectomy (OVX) and denervation. In this study, based on the fact that osteoclast differentiation and activation are mediated by RANKL, we used RANKL-treated mice as a model of bone loss. The mechanism of bone loss in this model is simple, in that excessive RANKL directly mediates the differentiation and activation of osteoclasts. The rapid decrease in bone mineral density (BMD) in this model seems not only to be caused by stimulation of the final differentiation of osteoclast progenitors but also to the activation of a preexisting pool of osteoclasts. However, the activation of osteoclasts by RANKL may be different from normal osteoclast activation by membrane-bound RANKL produced by osteoblasts. Osteoblast-bound RANKL would likely continue to stimulate osteoclasts by cell-to-cell interaction for longer than exogenous RANKL. The RANKL model is more protective of laboratory animal welfare because of the shorter experimental periods required, the lack of any requirement for anesthesia or surgery, and the lower numbers of treatments with test materials required compared with existing approaches. However, since the term osteoporosis refers to a specific form of bone-loss disease, we have avoided using this term in the title and elsewhere.

In this study, we hypothesize that simvastatin acts via IRF4 to suppress osteoclastogenesis. However, simvastatin is not an IRF4-specific inhibitor, and no IRF4 inhibitors have yet been developed. Simvastatin inhibits the numerous key proteins that function as molecular switches, including the small GTPases RAS, RAC and RAS homologue (RHO), and it is reported that RAS, RAC and RHO mediate osteoclastogenesis. Because of this, we cannot conclusively prove that simvastatin acts only via IRF4, which is one limitation of this study, but our findings strongly support our hypothesis concerning the role of IRF4 in osteoclastogenesis. Simvastatin suppresses osteoclastogenesis by inhibiting the expression of NFATc1 via the disappearance of IRF4. It was previously shown that the IRF-association domain (IAD) of IRF4 allows interaction with other IRFs such as IRF8 [12], [42] which suppresses osteoclastogenesis by inhibiting the function and expression of NFATc1 [15]. In contrast, in our study, IRF4 was not found to induce the association of IRF8 in osteoclastogenesis (data not shown). IRF8 has a suppressive role in TNF-α-induced osteoclastogenesis [15]. TNF-α stimulation involves activiation of the transcription factor nuclear factor-κB (NF-κB), which plays a crucial role in osteoclast differentiation. This report shows that the role of IRF8 is independent of NF-κB activation in osteoclast differentiation. The NF-κB inhibitor BAY11-7082, is one of the best-known osteoclastogenesis inhibitors, and is shown to reduce IRF4 protein levels in osteoclast differentiation (Fig. 3B). This result shows that the role of IRF4 is dependent on NF-κB activation in osteoclast differentiation. Thus, we hypothesize that the role of IRF4 and IRF8 are independent, and that the activity of the RANKL-regulated NFATc1 promoter is directly mediated by IRF4 in osteoclastogenesis.

We examined the mechanism underlying the increase in expression of IRF4 and NFATc1 with RANKL. The increase in NFATc1 and IRF4 expression and reduced H3K27me3 detection could be coincidental and not causal. De Santa *et al.* [43] have recently reported that Jmjd3 is activated in an NF-κB-dependent fashion, suggesting that therapeutic targeting of the NF-κB signalling pathway [44] may be rearranged by IRF4 signalling. Interestingly, in our study, the expression level of IRF4 mRNA was decreased the second day after RANKL treatment, in contrast to NFATc1 mRNA expression which continued to increase during osteoclastogenesis (Fig. 1D), and is induced by an established autoregulatory loop in which it binds to its own promoter region, leading to its robust induction [37]. By contrast, activation of EZH2-mediated H3K27 methylation increased during the later stage of osteoclastogenesis (Fig. 1A). Fig. 1B shows that EZH2-mediated H3K27 methylation increased on the promoter region of IRF4 and NFATc1 during the later stage of osteoclastogenesis. We believe that methylation acts to reduce IRF4 gene activation by the second day after RANKL stimulation.

**Figure 5:**
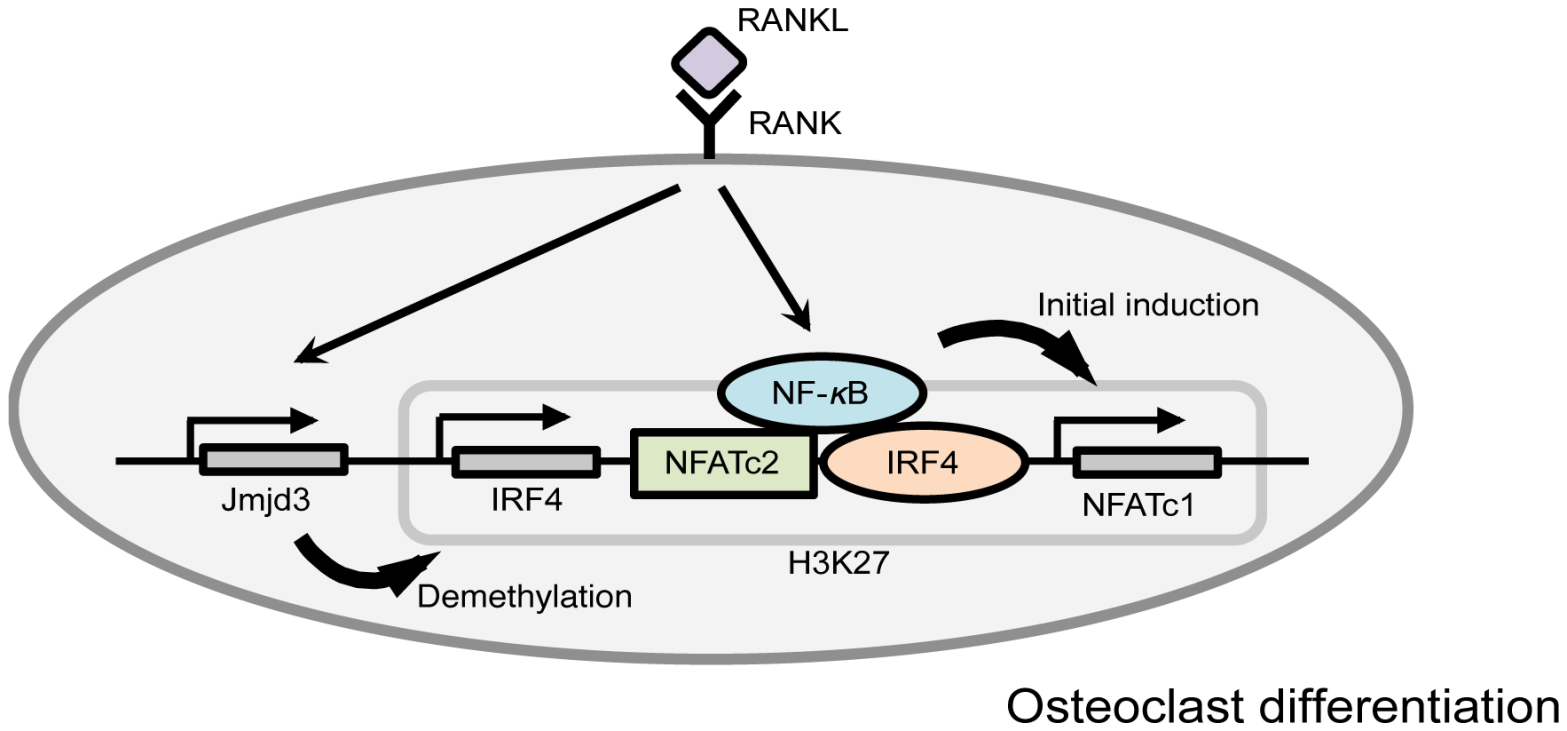
Model of osteoclastogenesis acceleration by IRF4. In osteoclast precursors, differentiation is regulated by epigenetic modification of the IRF4 and NFATc1 genes, and demethylation of H3K27me3 by Jmjd3 plays a critical role in this process. RANKL induces upregulation of IRF4, thereby augmenting IRF4 expression in the nucleus. We examined the mechanism of the increase in NFATc1 expression with RANKL. Stimulation of osteoclast precursors by RANKL results in activation of NF-κB which binds the NFATc1 promoter, cooperating with activated IRF4 and NFATc2 to induce initial induction of NFATc1. The increase in NFATc1 and IRF4 expression and decreased H3K27me3 detection could be coincidental and not causal.

Our data identify a mechanism by which IRF4 can enhance osteoclastogenesis (depicted in Fig. 5). A detailed analysis of the mouse NFATc1 promoter indicates that IRF4 can bind to DNA elements situated next to well-known NFATc1 binding sites, including autoamplification of its own promoter [45]. We further show that IRF4 can functionally cooperate with the NFATc1 protein and that the effect of IRF4 on expression of the osteoclastic genes *Atp6v0d2*, *Cathepsin K* and *TRAP* can be blocked by administration of simvastatin, which interferes with NFATc1 and IRF4 activation. Taken together these data are consistent with the notion that IRF4 can function as a lineage-specific partner for NFATc2 proteins [46]. Thus, the inductive effect of IRF4 upon osteoclast activation is likely to represent one of the critical steps that can endow osteoclasts with the ability to perform their unique set of biologic responses. Regarding formation of new bone and osteoblastic activity, performed toluidine blue staining and immunostaining of osteopontin, a key protein for the bone metabolism modulator which participates in bone formation and resorption. The present results demonstrated that in the statin group, the level of osteopontin and the volume of new bone were not affected by statin. And, Our results suggest that the depletion of osteoclast numbers were not due to the reduction in RANKL production by osteoblastic activation. Since we used RANKL-treated mice, the level of RANKL in bone rapidly increases. In an earlier report, it was demonstrated that mevastatin inhibited the fusion of osteoclasts and disrupted actin ring formation [47]. This finding is in accord with our results, because RANKL is an important protein for the fusion of preosteoclast cells [48]. Tumor necrosis factor alpha, interleukin-1, and interleukin-11 are also proteins which are well known to stimulate osteoclast differentiation. However, they act in a RANK/RANKL-independent manner [49]. To elucidate further the function of statins in osteoclast differentiation, a RANK/RANKL-independent osteoclast differentiation system should be examined in future studies.

In conclusion, this study provides evidence for the hitherto unknown effects of an IRF4 inhibitor (simvastatin) in inhibiting osteoclast differentiation and action, suggesting new therapeutic possibilities for the treatment of bone loss diseases.

## Supporting Information

Figure S1 **Full-length blots of** **Fig. 1** **.**
(TIF)

Figure S2 **Full-length blots of** **Fig. 2** **.**
(TIF)

Figure S3 **Full-length blots of** **Fig. 3** **.**
(TIF)
