# Supplementary figures and images for: Stimulation of Osteoclast Formation by RANKL Requires Interferon Regulatory Factor-4 and Is Inhibited by Simvastatin in a Mouse Model of Bone Loss
Source: PLoS One. 2013 Sep 11;8(9):e72033. doi: 10.1371/journal.pone.0072033 (PMC3770656; doi:10.1371/journal.pone.0072033)

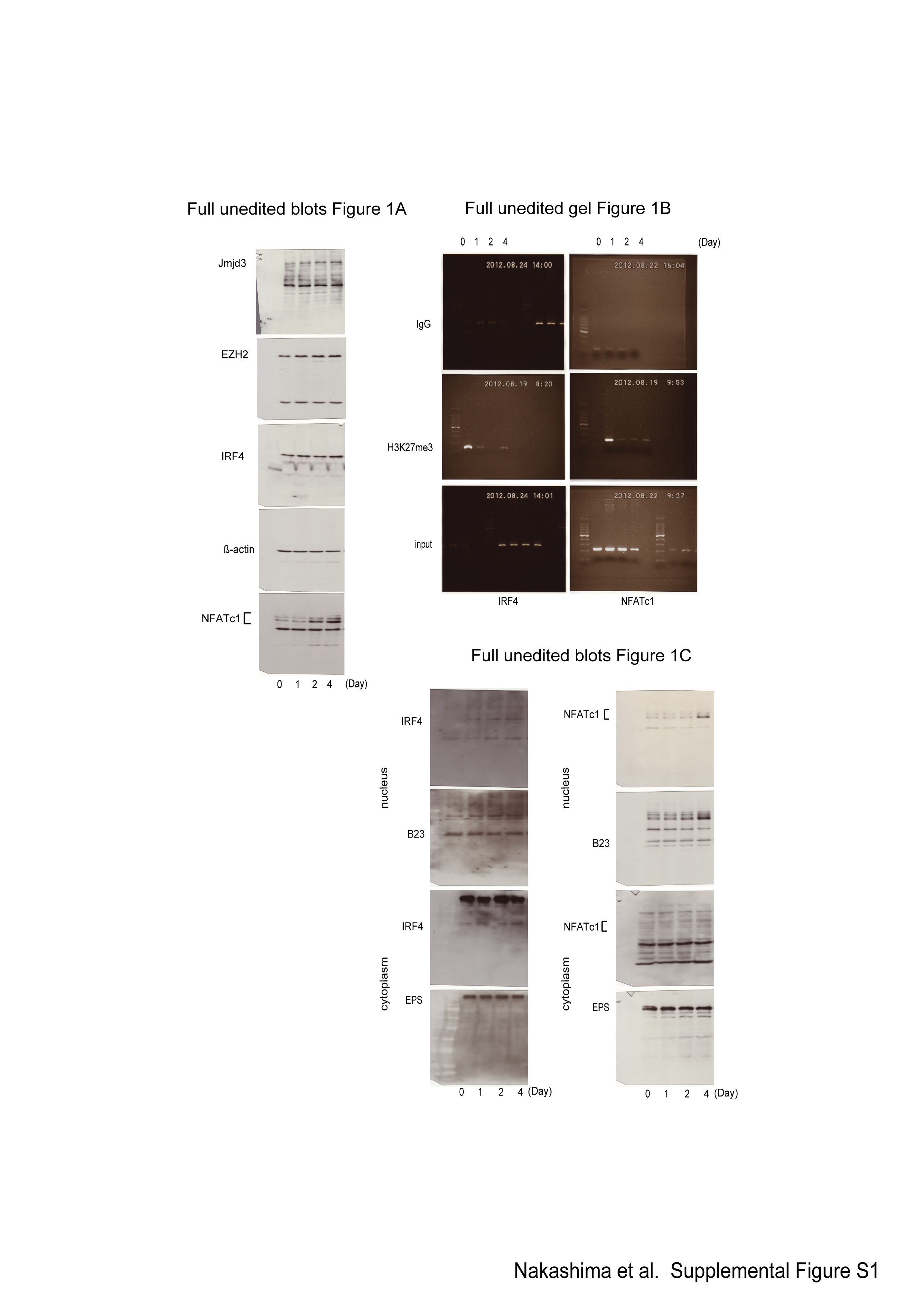

Supplement: Figure S1 — Full-length blots of Fig. 1 . (TIF) [file pone.0072033.s001.tif]

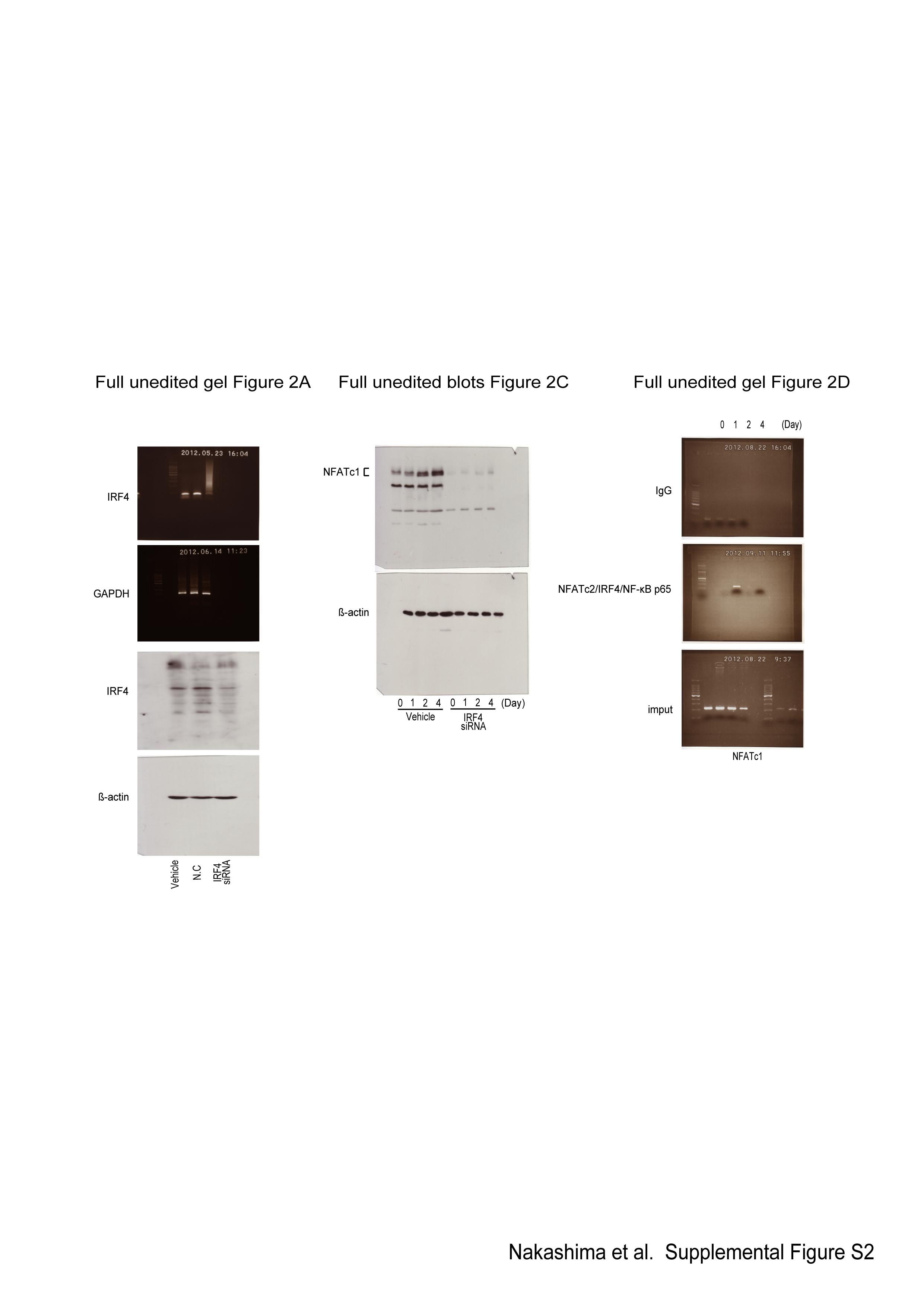

Supplement: Figure S2 — Full-length blots of Fig. 2 . (TIF) [file pone.0072033.s002.tif]

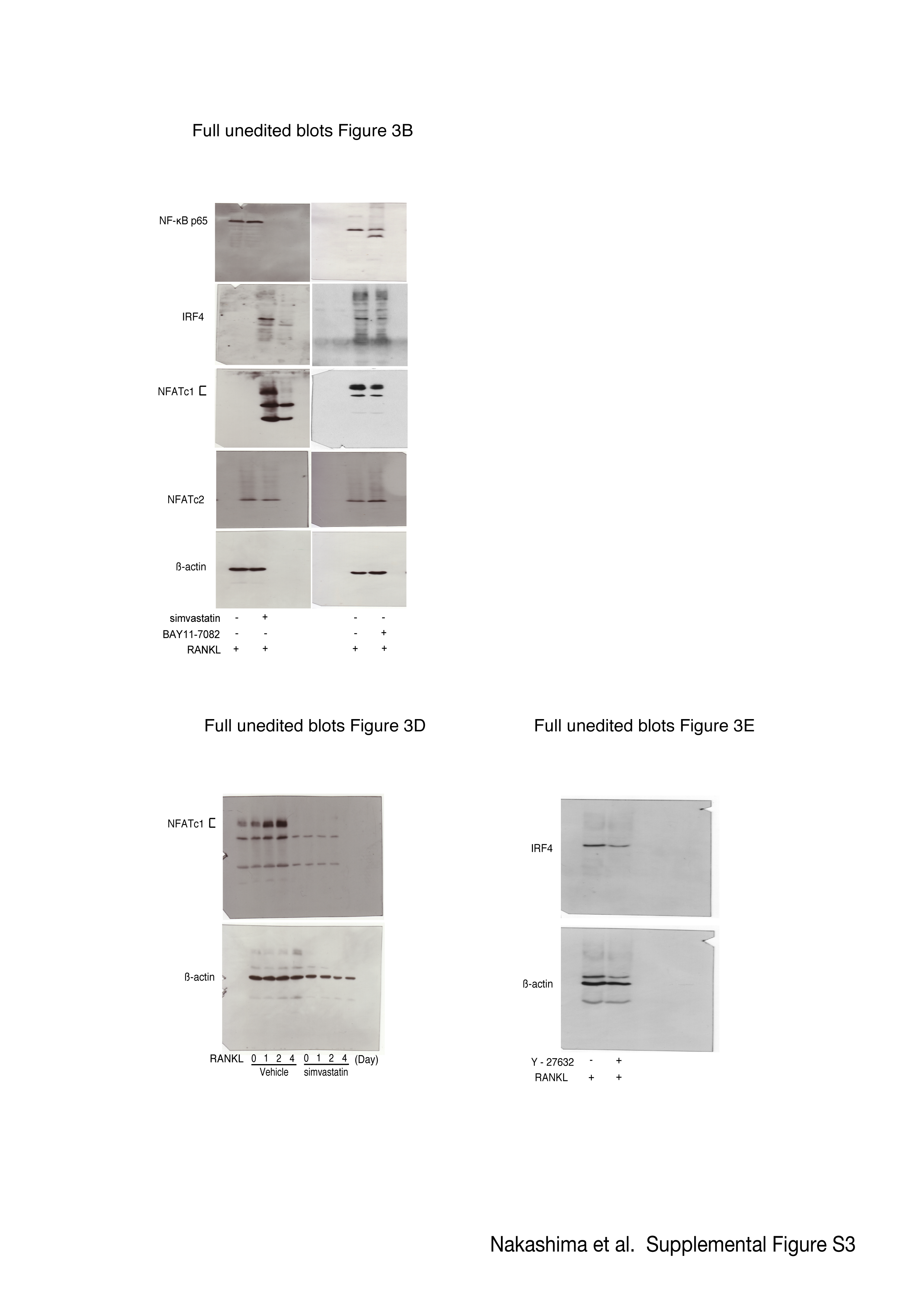

Supplement: Figure S3 — Full-length blots of Fig. 3 . (TIF) [file pone.0072033.s003.tif]
